# A randomized placebo-controlled phase I clinical trial to evaluate the immunomodulatory activities of *Atractylodes lancea* (Thunb) DC. in healthy Thai subjects

**DOI:** 10.1186/s12906-020-03199-6

**Published:** 2021-02-12

**Authors:** Inthuon Kulma, Luxsana Panrit, Tullayakorn Plengsuriyakarn, Wanna Chaijaroenkul, Siriprapa Warathumpitak, Kesara Na-Bangchang

**Affiliations:** 1 Graduate Program in Bioclinical Sciences, Chulabhorn International College of Medicine, Thammasat University (Rangsit Campus), Pathumthani, 12121 Thailand; 2 Center of Excellence in Pharmacology and Molecular Biology of Malaria and Cholangiocarcinoma, Thammasat University (Rangsit Campus), Pathumthani, 12121 Thailand; 3 Drug Discovery and Development Center, Office of Advanced Science and Technology, Thammasat University (Rangsit Campus), Pathumthani, 12121 Thailand

**Keywords:** *Atractylodes lancea*, β-Eudesmol, Atractylodin, Cholangiocarcinoma, Immunomodulatory activity

## Abstract

**Background:**

*Atractylodes lancea* (Thunb) DC. (AL) and bioactive compounds β-eudesmol and atractylodin have been demonstrated in the in vitro and in vivo studies for their potential clinical use in cholangiocarcinoma. The study was a randomized, double-blinded, placebo-controlled phase I clinical trial to evaluate the immunomodulatory effect of AL in human subjects.

**Methods:**

The modulatory effects of AL and β-eudesmol and atractylodin on TNFα and IL6 expression in PBMCs were measured using real-time PCR. Blood samples were collected from forty-eight healthy subjects following oral administration of a single or multiple dosing of capsule formulation of the standardized AL extract or placebo. Serum cytokine profiles, lymphocyte subpopulations (B lymphocytes, CD8^+^ cytotoxic T lymphocytes, CD4^+^ T-helper lymphocytes, and NK cells), and cytotoxic activity of PBMCs against the cholangiocarcinoma cell line CL-6 were evaluated using cytometric bead array (CBA) with flow cytometry analysis.

**Results:**

AL extract at almost all concentrations significantly inhibited both TNFα and IL6 expression in Con A-mediated inflammation in PBMCs. β-Eudesmol at all concentrations significantly inhibited only IL6 expression. Atractylodin at the lowest concentration significantly inhibited the expression of both cytokines, while the highest concentration significantly inhibited only IL6 expression. The administration of AL at a single oral dose of 1000 mg appeared to decrease IFNγ and IL10 and increase B cell, while significantly increase NK and CD4^+^ and CD8^+^ cells. A trend of increasing (compared with placebo) in the cytotoxic activity of PBMCs at 24 h of dosing was observed. AL at multiple dosing of 1000 mg for 21 days tended to decrease the production of all cytokines, while significantly inhibited IL17A production at 24 h of dosing. In addition, a significant increase in CD4^+^ and CD8^+^ cells was observed. A trend of increase in the cytotoxic activity of PBMCs was observed at 24 h but terminated at 48 h of dosing.

**Conclusions:**

The results confirm the immunomodulatory activity of AL in humans. This activity, in complementary with the direct action of AL on inducing cholangiocarcinoma cell apoptosis, suggests its potential role for CCA control.

**Trial registration:**

Retrospectively registered on 17 October 2020 [Thai Clinical Trials Registry (TCTR: www.clinicaltrials.in.th) Number TCTR20201020001#].

**Supplementary Information:**

The online version contains supplementary material available at 10.1186/s12906-020-03199-6.

## Background

Cholangiocarcinoma (CCA) is a fatal disease with the highest incidence in Southeast Asia, particularly Thailand [1]. Clinical efficacy and tolerability of the current chemotherapeutic drugs remain unsatisfactory. Development of alternative medicines, including those from natural sources is urgently needed. Several risk factors, *i.e,* bile duct disorders, liver diseases, digestive disorders, metabolic and endocrine disorders, and parasitic infections (*Opisthorchis viverrine* and *Clonorchis sinensis*) [1] cause chronic biliary inflammation and cholestasis, resulting in uncontrolled proliferation, genetic and epigenetic mutations and eventually malignant transformation and tumor formation [2]. The pro-inflammatory cytokines, i.e.*,* tumor necrosis factor-alpha (TNFα), interleukin (IL) 6 and 8 (IL6, IL8), and nitric oxide synthase (NOS) which are released during tissue injury and chronic inflammation of biliary tree, promote cholangiocarcinogenesis through different signalling pathways involved in cell growth, apoptosis, invasiveness, and angiogenesis [3]. TNFα and IL6 are pro-inflammatory cytokines that modulate the growth, differentiation and proliferation of various types of cells, including CCA [3]. The level of IL6 was found to be increased in serum and bile of patients with CCA and CCA cell lines [4, 5]. CCA tumor microenvironment plays a vital role in the regulation of tumor proliferation, angiogenesis, invasion, and preventing tumor cells from organismal immune reactions and apoptosis [6]. Tumor-associated macrophages (TAMs) are the most relevant infiltrating immune cell population within the tumor microenvironment [7]. TAMs modulate the CCA microenvironment by secreting TNFα, TGFβ (tumor growth factor-β), IL6, IL10, and VEGF-A (vascular endothelial growth factor-A), which support epithelial-to-mesenchymal transition (EMT), tumor growth, and metastasis [8].

The dried rhizome of *Atractylodes lancea* (Thunb) DC. (AL) has been used in Chinese (“Cang Zhu”), Japanese (“So-jutsu”), and Thai (“Khod-Kha-Mao”) traditional medicines for various pharmacological properties including anticancer, anti-inflammatory, antimicrobial activities, and activities on central nervous, cardiovascular, and gastrointestinal systems. Interestingly, the crude extract of AL and its bioactive compounds β-eudesmol and atractylodin have been demonstrated in a series of both in vitro and in vivo studies for their potential clinical use for CCA control [9]. The immunomodulatory activity of AL and its bioactive compounds in complementary with their direct action on inducing effects on CCA cell apoptosis [10–12] would support the potential role of AL for CCA control. The immunomodulatory activity of this plant, as well as some of its isolated compounds, has also been demonstrated [13]. The present study was a randomized, double-blinded, placebo-controlled phase I clinical trial to evaluate the immunomodulatory activity of AL in humans. The changes in the pro-inflammatory (IL2, IL4, IL6, IL17A, TNFα, and IFNγ) and anti-inflammatory (IL10) cytokine profiles, lymphocyte subpopulations, i.e., B lymphocytes, CD8^+^ cytotoxic T lymphocytes, CD4^+^ T-helper lymphocytes, and NK (natural killer) cells, and cytotoxic activity against CCA cell line, of peripheral blood mononuclear cells (PBMCs) from healthy subjects following the administration of a single and multiple dose regimens of the capsule formulation of the standardized AL extract were investigated. The confirmed immunomodulatory activity, in complementary with the direct action of AL on inducing CCA cell apoptosis,would support the potential of AL for further clinical development for CCA control in patients with early and late stage CCA.

## Methods

### In vitro evaluation of the modulatory effects on cytokine release by PBMCs

#### Preparation of stock solutions of test materials

The stock solutions of the test materials were prepared and stored at − 20 °C. The crude ethanolic extract of AL was dissolved with 50% (v/v) ethanol in distilled water to obtain 5 mg/ml stock solution. β-Eudesmol and atractylodin were dissolved with 100% ethanol to obtain 5 mg/ml stock solutions and further diluted with the complete medium (RPMI1640 supplemented with 10% fetal bovine serum albumin). The final concentration of ethanol in the working solution was lower than 0.1% (v/v).

#### Isolation of PBMCs

PBMCs were separated from blood samples within 6 h after collection using Ficoll-Paque™ (GE Healthcare, NJ, USA). In brief, the blood sample was diluted with 2x volume of 1x phosphate buffer saline (PBS, pH 7.4) and carefully layered over an equal volume of Ficoll-paque™ in a 15 ml-conical tube. The suspension was centrifuged (400×g, 30 min, 20 °C) and the upper layer was aspirated, leaving the mononuclear cell layer (lymphocytes, monocytes, and thrombocytes) undisturbed at the interphase. The mononuclear cell layer was transferred to a new conical tube, and 1x PBS was added. The supernatant was carefully removed following centrifugation (300×g, 10 min, 20 °C). Cell pellets were resuspended with 1xPBS. Following centrifugation (200×g, 10 min, 20 °C), the supernatant was carefully removed. The PBMC pellets were obtained through centrifugation over Ficoll–Paque™ cushions of buffy-coat, and cell number was counted using a cell counter. Cell suspension at a density of 5 × 10^6^ cells/ml was prepared in complete medium and incubated at 37 °C for 3 h.

#### Cytotoxic activity on PBMCs

PBMCs (100 μl of 2 × 10^5^ cells/well) were seeded onto a 96-well plate. Each test material was dissolved in complete medium. The final concentrations of the AL extract, β-eudesmol, and atractylodin were 500, 250, 125, 62.5, 31.25, 15.62, 7.81, and 3.95 μg/ml in the total volume of 100 μl/well. The plates were incubated at 37 °C under 5% CO_2_ for 24 h. Freshly prepared MTT [3-(4,5-dimethylthiazol-2-yl)-2,5-diphenyl tetrazolium bromide] reagent (5 mg in 20 μl PBS) was added into each well and further incubated for 4 h. Finally, the supernatant was removed, and DMSO reagent (100 μl) was added into each well with swirling to completely solubilize the formazan crystal. The absorbance was measured at 570 nm in a microplate reader (Vario skan flash, Thermo scientific, USA). The treated cells, untreated cells, and blank were assayed in triplicate for three times. The concentration-effect curve was analyzed, and the IC_50_ and IC_5_ (concentrations that inhibit cell growth by 50 and 5%, respectively) were determined using Calcusyn® version 1.1 (Biosoft, Cambridge, UK).

#### Exposure of PBMCs to standardized AL extract, β-eudesmol, and atractylodin

PBMCs (2 × 10^6^cells/well) were seeded onto a 6-well plate and exposed to standardized AL extract, β-eudesmol, and atractylodin at the final concentrations of 40, 20 and 10 μg/ml. Concanavalin A (ConA, 10 μg/ml) and dexamethasone (200 μM) were, respectively, used to induce and inhibit (positive control) cell inflammation. To investigate the combined effect of the three test materials, cells were exposed to the standardized AL extract, β-eudesmol, and atractylodin at the final concentrations of 40, 20 and 10 μg/ml, together with 10 μg/ml of ConA. The untreated cell served as a negative control. The plates were incubated at 37 °C under 5% CO_2_ for 24 h, and the cells were collected through centrifugation at 250×g for 10 min. The experiment was repeated three times, triplicate each.

#### Extraction of RNA

The RNA content of the exposed PBMCs was extracted with TRIzol™ (ThermoFisher Scientific, MA, USA). Briefly, PBMCs were added with 50 μl of chloroform and transferred to a homogenizer (Scilogex D500, CT, USA). The homogenate was mixed and centrifuged at 12,000×*g* for 15 min (4 °C). The supernatant was separated and transferred to a new tube. Cold isopropanol (200 μl) was added, mixed, and centrifuged at 12,000 x *g* for 15 min (4 °C). The remaining supernatant was discarded, and the RNA pellets were washed with 1 ml of 100% cold ethanol and left to dryness. The RNA pellets were dissolved with 20 μl of DEPC water, and total RNA content was measured at 260 nm by spectrophotometer (ThermoFisher Scientific, MA, USA).

#### Synthesis of cDNA

The cDNA was prepared using the SuperScript® III First-Strand Synthesis System for RT-PCR according to the manufacturer’s protocol. Briefly, 2 μl of RNA from PBMCs (500 ng/ml), 0.5 μl of 50 μM oligo (dT), and 10 μM of dNTP mix were mixed and incubated at 65 °C for 5 min. The mixture was immediately plated on ice for at least 1 min. The cDNA synthesis mixture was prepared by adding 2 μl of 10x RT buffer, 4 μl of 25 mM MgCl_2,_ 2 μl of 0.1 M DTT, 1 μl of RNase OUT (40 U/μl), and 1 μl of SuperScript™ III RT (200 U/μl). Ten μl of the cDNA synthesis mixture was added into each RNA template and incubated at 50 °C for 50 min. The reaction was terminated by incubation at 85 °C for 5 min. RNase H (1 μl) was added into each tube and incubated at 37 °C for 20 min. The synthesized cDNA was stored at − 20 °C until use.

#### Quantification of mRNA expression of interleukin 6 (IL6) and tumor necrosis factor-alpha (TNFα)

The levels of IL6 and TNFα were quantified using iTaq™ Universal SYBR™ Green Supermix (Bio-Rad Laboratories Inc., CA, USA). The mRNA expression level of IL6 and TNFα were normalized with glyceraldehyde-3-phosphate dehydrogenase (GAPDH). Amplification of cDNA was performed on Bio-Rad CFX (Bio-Rad, CA, USA) using the following sequences of IL6 and TNFα forward and reverse primers (Table 1). The real-time PCR conditions consisted of denaturing at 95 °C for 10 s, annealing at 60 °C for 10 s, and extension at 72 °C for 20 s (49 cycles). Each analysis was performed in three independent experiments, triplicate each. The delta-delta Ct calculation for the relative quantification of the target gene was as follow:

$$ \Delta \mathrm{Ct}\ (1)=\left[\mathrm{Ct}\left(\mathrm{IL}-6\ \mathrm{or}\ \mathrm{TNF}-\upalpha \right)-\mathrm{Ct}\left(\mathrm{GAPDH}\right)\right] $$

$$ \Delta \mathrm{Ct}(2)=\Big[\mathrm{Ct}\left(\mathrm{control}\ \mathrm{for}\ \mathrm{IL}-6\ \mathrm{or}\ \mathrm{TNF}-\upalpha \right)-\mathrm{Ct}\left(\mathrm{control}\ \mathrm{for}\ \mathrm{GAPDH}\right) $$

$$ \Delta \Delta \mathrm{Ct}=\Delta \mathrm{Ct}(1)-\Delta \mathrm{Ct}(2) $$

$$ \mathrm{Relative}\ \mathrm{expression}={2}^{-\Delta \Delta \mathrm{Ct}} $$

**Table 1:** Primer sequences for the real-time PCR experiment

Gene name	Forward primer	Reverse primer
IL-6	GTACATCCTCGACGGCATC	AGCCACTGGTTCTGTGCCT
TNF-α	TGCTTGTTCCTCAGCCTCTT	ATGGGCTACAGGCTTGTCACT
GAPDH	TCAACGGATTTGGTCGTATT	CTGTGGTCATGAGTCCTTCC

Where ΔCt (1) = delta Ct of unknown sample, ΔCt (2) = delta Ct of control, IL-6 or TNF-α = target gene, and GAPDH = housekeeping gene.

### Ex vivo evaluation of the effects on cytokine production and immune cell activity

#### Participants and study design

The study was an open, randomized, double-blinded, placebo-controlled design, conducted at Clinical Research Center, Faculty of Medicine, Thammasat University. Approval of the study protocol was obtained from the Ethics Committee, Thammasat University. The written informed consent was obtained from all research participants. The study protocol including background and rationale of the study, methodology, information about the results of non-clinical studies of AL from previous studies particularly the safety profile, as well as the risks and benefits of study participation was explained to all potential participants in details. A total of 48 healthy Thai participants (24 males and 24 females), aged between 20 and 45 years with body mass index (BMI) between 20 and 25 kg/m^2^, who were non-smokers and non-alcohol drinkers and were residents of Bangkok or suburb areas were recruited into the study. The sample size included in the study was based principally on the primary pharmacokinetic outcome parameters. Additional inclusion criteria were (i) absence of acute or chronic diseases that could affect vital organ functions, (ii) no history of surgery within the past six months, (ii) no history of hypersensitivity reactions or idiosyncratic reactions to drugs or herbal products, (iv) no concurrent or history of administration of drugs or herbal products within the past two weeks (except antipyretic or anti-emetic drugs), (v) no history or current drug abuse, (vi) ability to communicate (reading, writing, and speaking) effectively, and (vii) willing to give informed consent for study participation. Exclusion criteria included those with (i) clinical significant abnormality of physical examination, (ii) clinical significant abnormality of electrocardiograms (ECG) or chest x-ray, (iii) pregnancy or lactation, (iv) blood tests positive for HBsAg, HCV, or HIV, (v) abnormality in blood coagulation or history or concurrent use of anticoagulants or antiplatelets, or (vi) participation in any other study in the past three months. The exclusion of each potential participant from the study was confirmed through medical and laboratory records and verbal explanation.

After obtaining written informed consent, clinical and laboratory investigations were carried out to confirm the eligibility of the research participants. These included physical examination, electrocardiogram (ECG) monitoring, chest X-ray test, and laboratory investigations (hematology, serum biochemistry, blood coagulation, urinalysis, serology, and pregnancy status). Eligible participants were admitted to the ward at the Clinical Research Center, Faculty of Medicine, Thammasat University during the first two days of the pharmacokinetic study and returned for drug administration and follow up daily until 12 or 21 days, depending on the allocated drug regimens.

#### Drug administration and blood sample collection

Study participants were allocated to two groups (12 males and 12 females for each group) with equal age distribution [median (range) ages for group 1 and group 2 were 22.0 (21.0–27.0) and 23.0 (21.0–30.0) years, respectively] as follows:

- Group 1: Participants were randomized (using a randomization table generated by the statististician) to receive a single oral dose of either 1000 mg of capsule formulation of the standardized AL extract (9 capsules, 112.5 mg each, Kaolaor Laboratories Co. Ltd.) or placebo at the ratio of 20:4 participants. EDTA blood samples (20 ml for each time point) were collected at 0, 24 (day 1), 48 (day 2) hours, and days 7 and 14 of dosing.
- Group 2: Participants were randomized to receive multiple oral doses of either 1000 mg of capsule formulation of the standardized AL extract or placebo daily for 21 days at the ratio of 20:4 participants. Blood samples (20 ml for each time point) were collected at 0, 24 (day 1), 48 (day 2) hours of the first dose on day 1, and before dosing on days 7, 14, 21, and 22.

All capsules were taken at once with 200 ml drinking water. No food was consumed, although alcohol-free and xanthine-free fluids (fluids that contain caffeine, which has been reported for anti-inflammatory and inhibitory effect on immune response) were permissible the night before the study. Participants were fasted for 2 h after drug administration to avoid any interaction between food and drugs. No other drugs, except analgesic, antipyretic and anti-emetic drugs were allowed during the study period to avoid possible interference of the immunomodulatory activity of these concurrent medications with the study drug AL.

Three millilitres of blood were used for measurement of lymphocyte subpopulations, and 12 ml were used for evaluation of the cytotoxic activity. Clotted blood (5 ml) samples were collected for the investigation of cytokine levels.

#### Determination of cytokine levels

Cytokine bead array (CBA) Th1/Th2/Th17 cytokine kit (BD Biosciences, USA) was used to measure the levels of the secreted cytokines in serum samples according to the manufacturer’s protocols (duplicate for each sample). Cytokine standards (IL17A, IFNγ, TNFα, IL2, IL4, IL6, and IL10) were prepared by reconstituting human Th1/Th2/Th17 cytokine standards in 0.2 ml of assay diluent to prepare 10x bulk standards. The standards were diluted in the assay diluents to obtain the dilutions of 1:2, 1:4, 1:8, 1:16, 1:32, 1:64, 1:128, and 1:256. The suspension was mixed and used at 10 μl/test, and 50 μl of mixed beads were transferred to each assay tube. Each standard cytokine dilution or unknown sample (50 μl) and PE Detection Reagent (50 μl) were added. The mixture was incubated at room temperature for 3 h in the dark, and washed with wash buffer (1 ml), and centrifuged at 200×g for 5 min. Finally, the wash buffer (300 μl) was added for further flow cytometry analysis. Cytometer setup bead tube used for flow cytometer setup before sample analysis was prepared by adding 100 μl of cytometer setup beads from the BD CBA kit and 400 μl of wash-buffer and thoroughly mixed. The level of each cytokine was normalized with total white blood cell count of each subject.

#### Measurement of T-lymphocyte subpopulations

EDTA-blood samples were stained with BD Multitest 6-color TBNK kit™ (lymphocyte subpopulations: B cells, CD8^+^cytotoxic T lymphocytes, CD4^+^T-helper lymphocytes, and NK cells) and analyzed by Facsverse™ flow cytometer. Blood samples were stained with BD Multitest 6-color TBNK kit™ (BD, Biosciences, USA). In brief, 20 μl of BD Multitest 6-color TBNK reagent was added into the bottom tube with 50 μl of EDTA anti-coagulated blood, gently mixed, and incubated for 15 min in the dark at room temperature (25 °C). Following incubation, 450 μl of 1xBD FACS lysing solution (BD, Biosciences, USA) was added, gently mixed, and incubated for 15 min in the dark at room temperature. The number of immune cells was determined using flow cytometry (BD Pharmingen, NJ, USA). The number of all T-lymphocyte subpopulations was normalized with total white blood cell count of each subject.

#### Investigation of cytotoxic activity of PBMCs

The cytotoxic activity against the CCA cell line CL-6, of the PBMCs isolated from blood samples collected from healthy subjects after administration of AL extract and placebo in both groups were evaluated using flow cytometry-based NK cytotoxicity assay [14].

#### Preparation of effector cell

PBMCs were used as effector cells. The cells were separated from blood samples of healthy volunteers within 6 h after collection using histopaque-1077 (Sigma, St. Louis, USA) according to the manufacturer’s instructions. Histopaque-1077 was added into a 50-ml conical centrifuge tube and carefully layered with the whole blood samples. The samples were centrifuged at 400×g for 30 min at room temperature. The opaque interface containing mononuclear cells was transferred to a clean conical centrifuge tube. The cells were washed twice by adding 10 ml and 5 ml of isotonic phosphate-buffered saline solution, followed by centrifugation at 250×g for 10 min. The supernatant was discarded, and the cell pellets were resuspended with freezing media (90% FBS and 10% DMSO), placed in a Nalgene Cryo 1 °C freezing container, frozen at − 80 °C, and then transferred to liquid nitrogen storage until use. The day prior to assay, cryopreserved PBMC samples were thawed quickly in a 37 °C water bath, washed twice in complete RPMI-1640 medium and cell number was counted. Samples were resuspended in complete medium containing Antibiotic-Antimycotic (100 U/ml) solution and were maintained in humidified conditions (5% CO_2_, 37 °C) in a CO_2_ incubator (HERA CELL 150i, Thermo Scientific, MA, USA) for 24 h. The effector cells were stained with 0.05 μM CAM in 10 ml of complete medium for 30 min. Cells were washed twice in complete medium and used in the flow cytometry-based NK cytotoxicity assay.

#### Preparation of target cell

The human CCA cell line CL-6 (a gift from Associate Professor Adisak Wongkajornsilp, Faculty of Medicine, Siriraj Hospital, Thailand) was used to evaluate the cytotoxic activity of the prepared PBMCs. The cell was cultured in complete medium containing Antibiotic-Antimycotic (100 U/ml) solution and maintained in humidified conditions (5% CO_2_, 37 °C) in a CO_2_ incubator (HERA CELL 150i, Thermo Scientific, MA, USA). The cultured cells of 80% confluence were harvested using 0.25% Trypsin-EDTA.

#### Flow-cytometry-based NK cytotoxicity assay

CAM-stained effector cells (PBMCs) were seeded in triplicate with a fixed number of unstained naïve target cells (10,000/ well) onto a 96-well microtiter plate with 50:1 (E:T ratios). Additional wells were used for the assessment of spontaneous apoptosis (target cells only in 200 μl of complete medium) and maximum target cell death (target cells only in 100 μl of complete medium plus 100 μl of 100% DMSO). The contents of each well-containing effector and target cells were incubated for 1 h at 37 °C in a humidified incubator under a 5% CO_2_ atmosphere. The mixed cells (in 200 μl of complete medium) were transferred to a new tube and washed twice with PBS. Five to 10 min before the acquisition, 10 μl of 1 μg/ml propidium iodide (PI) (Sigma-Aldrich, St. Louis, USA) was added to each tube. Flow cytometry was performed using a BD FACSVerse flow cytometer (BD Biosciences, San Jose, USA). At least 5000 target cells *per* sample were acquired in order to determine the lysis of target cells by flow cytometry. To calculate net lysis of target cells, the PI-positive target cells in medium alone was subtracted from that of each sample.

### Statistical analysis

Statistical analysis was performed using SPSS for Windows Software version 12 (IBM, New York, USA). The nonparametric analysis was applied for non-normally distributed data. Quantitative data are summarized as median (range), and qualitative data are summarized as number and percentage (%) values. Comparison of two independent quantitative variables was performed using the Mann-Whitney U test. Statistical significance level was set at α = 0.05 for all tests.

## Results

### In vitro evaluation of the modulatory effects on cytokine release by PBMCs

The cytotoxic effects, expressed as IC_50_ [median (range)], of the crude ethanolic extract of AL, atractylodin and β-eudesmol on PBMCs were 147.06 (121.12–152.34), 134.31 (125.32–142.54) and 139.26 (125.87–150.22) μg/ml, respectively. The corresponding IC_5_ values were 41.32 (38.33–43.01), 39.38 (38.32–42.28) and 40.66 (38.71–42.04) μg/ml, respectively. The highest concentration of AL, atractylodin and β-eudesmol that resulted in 5% of cell growth inhibition (IC_5_) of about 40 μg/ml, and the two lower concentrations (20 and 10 μg/ml) were therefore selected as the highest concentration used in further RNA analysis of TNFα and IL6 expression in PBMCs. AL extract at all concentrations except at the lowest concentration (induces TNFα expression) significantly inhibited both TNFα and IL6 expression in Con A-mediated inflammation in PBMCs with strong potency. β-Eudesmol at all concentrations significantly inhibited only IL6 expression with strong potency. Atractylodin at the lowest concentration significantly inhibited the expression of both cytokines, while the highest concentration significantly inhibited only IL6 expression (Table 2).

**Table 2:** The immunomodulatory effects (expressed as fold-changes compared with untreated control) of AL extract, β-eudesmol, and atractylodin at the concentrations of 40, 20 and 10 μg/ml, on the expression levels of TNFα and IL6 in PBMCs compared with untreated control cells. Concanavalin A (Con A) alone (10 μg/ml) and dexamethasone (200 μM) were used to stimulate and inhibit (positive control) the inflammatory process in PBMCs. Data are presented as median (range) values of three independent experiments (triplicate each)

Treatment	Fold changes	
	TNFα	IL6
Untreated cells	1.00	1.00
Con A (10 μg/ml)	**2.19 (1.99–2.23)**	**1.02 (0.99–1.05)**
Dexamethasone (200 μm) + Con A (10 μg/ml)	0.60 (0.59–0.61)^b^	0.33 (0.32–0.33)^b^
AL (40 μg/ml) + Con A (10 μg/ml)	0.99 (0.98–1.00)^b^	0.01 (0.009–0.012)^a^
AL (20 μg/ml) + Con A (10 μg/ml)	1.65 (1.60–1.67)^c^	0.12 (0.11–0.12)^a^
AL (10 μg/ml) + Con A (10 μg/ml)	2.5 (2.49–2.51)	0.45 (0.43–0.49)^b^
β -eudesmol (40 μg/ml) + Con A (10 μg/ml)	2.26 (2.24–2.27)	0.35 (0.34–0.36)^b^
β -eudesmol (20 μg/ml) + Con A (10 μg/ml)	2.2 (2.21–2.23)	0.58 (0.56–0.58)^c^
β -eudesmol (10 μg/ml) + Con A (10 μg/ml)	2.45 (2.31–2.54)	0.70 (0.69–0.71)^c^
Atractylodin (40 μg/ml) + Con A (10 μg/ml)	3.46 (3.20–3.54)	0.77 (0.74–0.80^c^
Atractylodin (20 μg/ml) + Con A (10 μg/ml)	1.96 (1.91–2.11)	1.94 (1.90–2.00)^d^
Atractylodin (10 μg/ml) + Con A (10 μg/ml)	0.26 (0.23–0.26)^a^	0.11 (0.09–0.12)^a^

^***a***^ *Statistically significant difference with Con A (P < 0.0001, Mann-Whitney U test)*

^***b***^ *Statistically significant difference with Con A (P < 0.001, Mann-Whitney U test)*

^***c***^ *Statistically significant difference with Con A (P < 0.01, Mann-Whitney U test)*

^***d***^ *Statistically significant difference with Con A (P < 0.05, Mann-Whitney U test)*

### Ex vivo investigation of the effects on cytokine production and immune cell activity

**Fig. 1:**
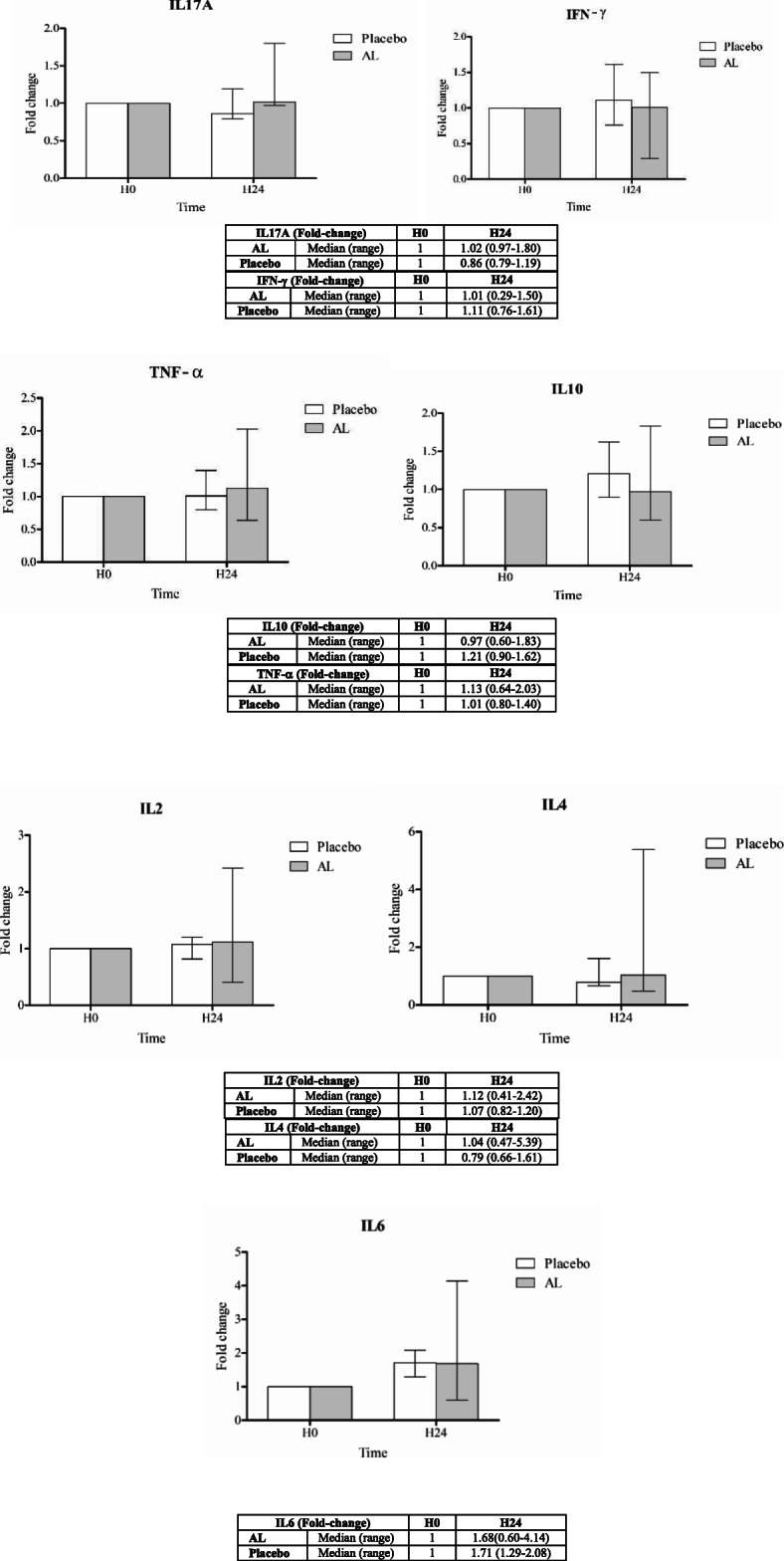
Fold-changes of IL17A, IFNγ, TNFα, IL2, IL4, IL6, and IL10 compared with baseline levels in healthy Thai subjects following the administration of a single oral dose of 1000 mg of standardized AL extract (*n* = 20) and placebo (*n* = 4). Data are presented as median (range) values

**Fig. 2:**
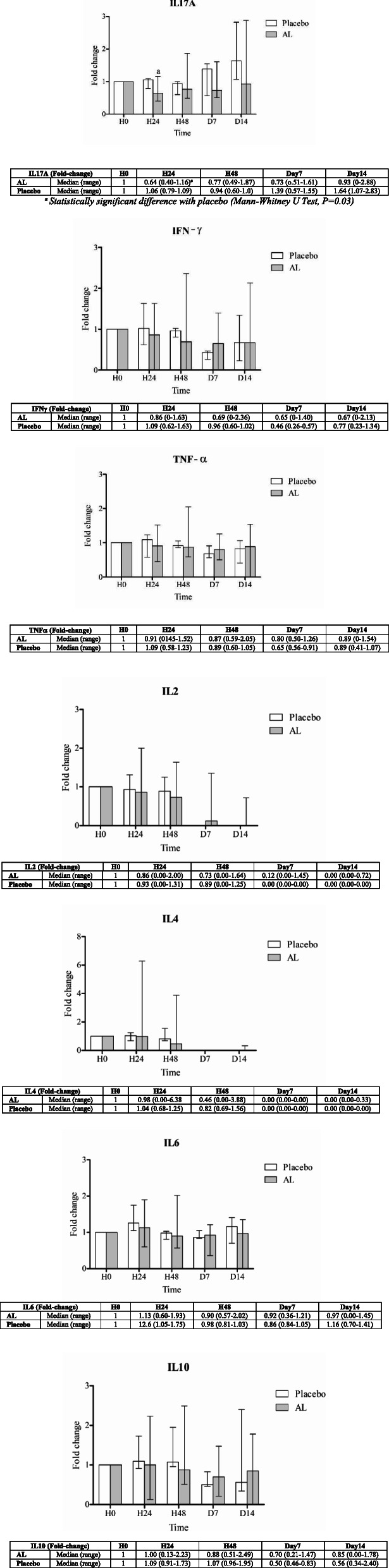
Fold-changes of IL17A, IFNγ, TNFα, IL2, IL4, IL6, and IL10 compared with baseline levels in healthy Thai subjects following the administration of daily oral doses of 1000 mg standardized AL extract (n = 20) and placebo (n = 4). Data are presented as median (range) values

Ex vivo investigation of the effects on cytokine production and immune cell activity was performed using blood samples collected from all 48 subjects at specifiedtime points. All subjects were healthy as verified by clinical and laboratory assessments (Supplementary file). The production of IL17A, IFNγ, TNFα, IL2, IL4, IL4, and IL10 cytokines in serum samples (expressed as fold-change compared with baseline level) from healthy subjects following the administration of a single (*group 1*) and multiple (*group 2*) dosing of 1000 mg standardized AL extract and placebo is summarized in Figs. 1 and 2, respectively. For a single oral dose, a trend of increasing cytokine production (compared with placebo) at 24 h of dosing was observed with IL17A, TNFα, IL2, and IL4, while a trend of decreasing production was observed with IFNγ and IL10. The level of IL6 was comparable between the two groups (Fig. 1). For multiple oral dosing, a trend of decreasing the production of all cytokines was observed at 24 and 48 h of dosing, but significant difference was found only with IL17A at 24 h of dosing. The production of most cytokines (IFNγ, TNFα, IL6, and IL10) were increasing on days 7 and 14 (Fig. 2).

**Fig. 3:**
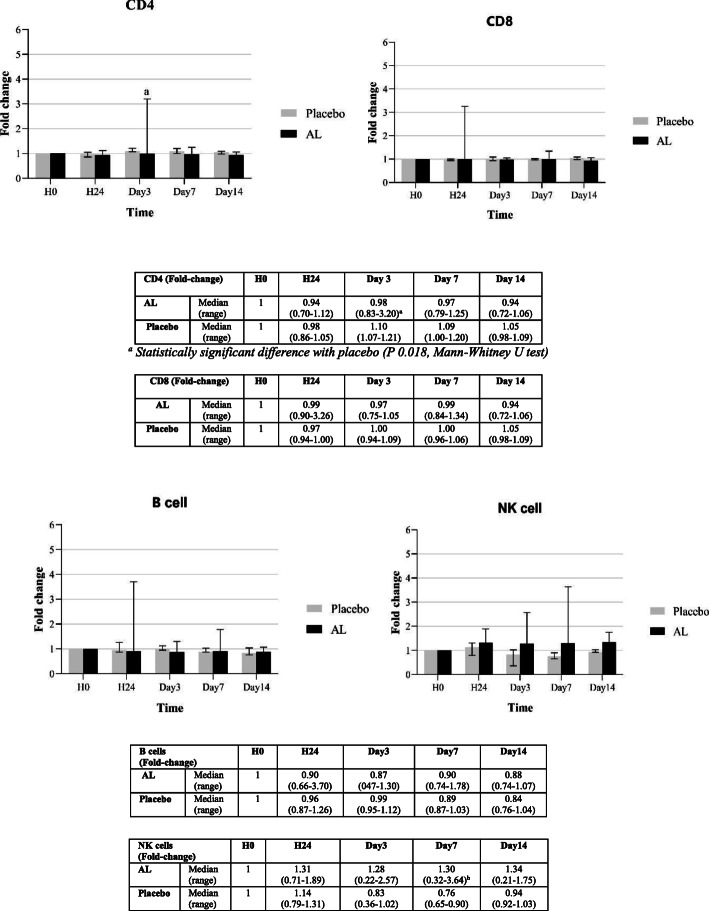
Fold-changes of CD4^+^, CD8^+^, B and NK cells compared with baseline levels in blood samples from healthy Thai subjects following the administration of a single oral dose of 1000 mg standardized AL extract (*n* = 16) and placebo (n = 4). Data are presented as median (range) values

**Fig. 4:**
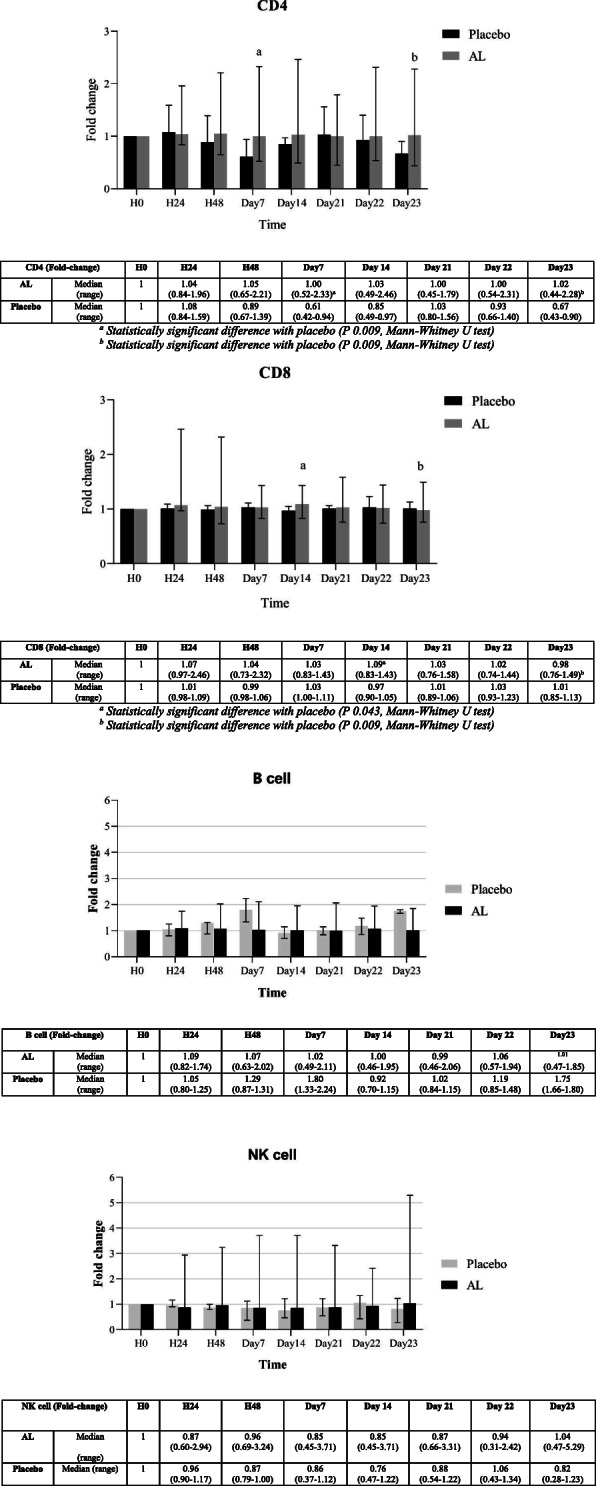
Fold-changes of CD4^+^, CD8^+^, B and NK cells compared with baseline levels in blood samples from healthy Thai subjects following the administration of daily oral doses of 1000 mg standardized AL extract (n = 20) and placebo (n = 4). Data are presented as median (range) values

The modulatory effects (expressed as fold-change compared to baseline) of AL on the production of various subtypes of lymphocyte subpopulations in blood samples from both groups of subjects are summarized in Figs. 3 and 4, respectively. For a single oral dose of AL, a significant increase in NK cells (compared with placebo) was observed at almost all time points (days 3, 7, and 14), while a trend of increasing in B cells was observed on days 1, 7, and 14 (Fig. 3). For multiple dosing, a significant increase in CD4^+^ was observed on days 7 and 23, and a significant increase in CD8^+^ was observed on day 14 (Fig. 4).

**Fig. 5:**
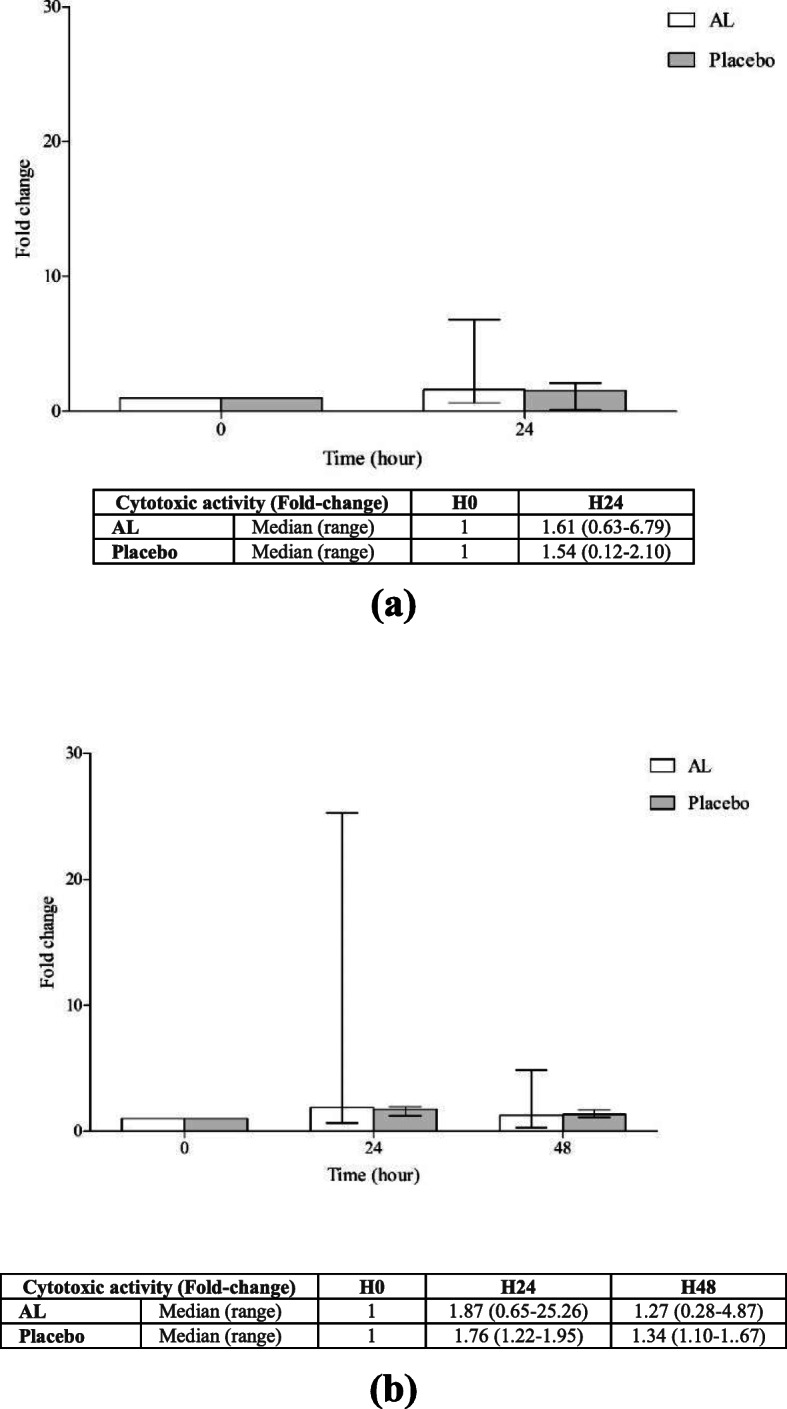
Fold-changes of cytotoxic activity of PBMCs against CL-6 cell compared with baseline levels in blood samples from healthy Thai subjects following the administration of (**a**) single oral dose of 1000 mg and (**b**) multiple oral doses of 1000 of standardized AL extract for 21 days (n = 20 for AL and n = 4 for each group). Data are presented as median (range) values

The modulatory effect (expressed as fold-change compared to baseline) of AL on the cytotoxic activity of NK cells in PBMCs (E:T ratio, 50:1) against CL-6 cell obtained from healthy subjects following the administration of the standardized AL extract (*n* = 20) and placebo (*n* = 4) is summarized in Fig. 5 (a,b). For a single dose, a trend of increasing (compared with placebo) in the cytotoxic activity of PBMCs at 24 h of dosing was observed (Fig. 5a). For multiple dosing, a trend of increasing in the cytotoxic activity of PBMCs was observed at 24 h but terminated at 48 h of dosing (Fig. 5a).

## Discussion

Inflammation is a protective response to eliminate harmful stimuli and immune cells are the major participants in this process [15]. During inflammation, bone marrow-derived monocytes are recruited to the site and differentiate into macrophages [16]. Macrophages eliminate pathogens and antigens through phagocytosis and induce inflammatory responses by producing cytokines and enzymes such as TNFα, IL6, inducible nitric oxide synthase (iNOS), and cyclooxygenase-2 (COX2) [17]. Inflammatory cytokines are generally classified as pro-inflammatory (IL1, IL4, IL6, IL12, IL15, IL17, IL23, TNFα, and IFNγ) or anti-inflammatory (IL10 and TGFβ) [18, 19]. TNFα and IL6 are pro-inflammatory cytokines that modulate the growth, differentiation and proliferation of various types of cells, including CCA [3]. IL6 activates the p38 and p44/42 MAPK pathway, leading to the downregulation of p21^WAF/CIP1^, a key gene regulator of cell cycle and anchorage-dependent cells growth [20]. Overexpression of IL6 reduces methylation of the EGFR promoter and enhances EGFR expression, leading to tumor growth promotion [21]. In addition, IL6 upregulates Mcl-1 (an apoptosis inhibitor) enhancing tumour cells survival by increasing expression of STAT3 [22]. TNFα induces immune response at tissue injury locations, and thus, the secretion of oxidative radicals such as hydroxyl radical, NO, and superoxide anion. These molecules are associated with aggressive development of CCA by causing DNA damage and inhibiting DNA repair proteins [23]. TNFα promotes CCA cell migration by upregulating the expression of the EMT markers S100A4, vimentin, and ZEB2 [24]. It is proposed that anti-TNFα therapy could be an effective approach for CCA [25]. IL6 [26] and TNFα [27] promote tumor angiogenesis through increasing vascular endothelial growth factor (VEGF) expression. Increase in the production of both pro-inflammatory (IFNγ, IL1β, IL2, IL4, IL5, IL6, IL8, IL12p70, TNFα, and LTα) and anti-inflammatory (IL10) cytokines in PBMCs were reported in *O. viverrini*-associated human CCA compared to uninfected control [28]. In the present study, AL extract at all concentrations except the lowest concentration (induced TNFα expression) significantly inhibited both TNFα and IL6 expression in Con A-mediated inflammation in PBMCs with strong potency. β-Eudesmol at all concentrations significantly inhibited only IL6 expression with strong potency. Interestingly, the lowest concentration of atractylodin significantly inhibited the expression of both cytokines, while the highest concentration significantly inhibited only IL6 expression.

In a previous study, AL was shown to reduce the inflammatory mediators TNFα and IL6 [29]. Additionally, atractylodin was reported to reduce the production of TNFα, IL1β, IL4, IL6, and CSF2 [30] in the stimulated human mast cell (HMC-1). In the animal model, AL exerted antigastric ulcer activity through suppression the pro-inflammatory production mediators TNFα, IL6, IL8, and prostaglandin E2 (PGE2) [29]. Other isolated compounds such as atractylenolide I, III, and atractylone were reported to exert anti-inflammatory activities. Atractylenolide I down-regulated the release of TNFα, IL6, IL1β, and IL13 [31]. The release of the pro-inflammatory cytokines stimulated by TSLP (TNFα, IL6, and IL8) was also reduced by atractylenolide III [32]. Moreover, the compound also inhibited the release of NO, TNFα, PGE2, and IL6 [33]. Atractylone reduced IL4 [34], IL6 [34, 35] and TNFα [35] levels. β-eudesmol was reported to inhibit the production and expression of IL6 through activation if p38 MAPK, NK-κB and caspase-1 in activated human mast cells [36]. Besides, it also reduced serum levels of histamine, IgE, IL1b, IL4, IL5, IL13 and VEGF in the passive anaphylaxis mouse model [37]. Altogether, the results of this study and previous studies suggested that the immunomodulatory effect of AL could be sum of the activity of each component in AL. Further analysis of the ex vivo immunomodulatory effect of AL was therefore performed in healthy subjects after oral administration of single or multiple doses of the standardized AL extract. Results indicated a trend of increasing of serum cytokine levels, i.e., TNFα, IL2, IL4, and IL17A at 24 h after a single oral dose of 1000 mg AL. A trend of decreasing cytokine production was, on the other hand, observed with IFNγ and IL10. For multiple oral dosing, a trend of decreasing in the production of all cytokines was observed at 24 and 48 h of dosing, but significant difference was found only with IL17A at 24 h of dosing. On days 7 and 14, the production of most cytokines (IFNγ, TNFα, IL6, and IL10) were increasing. IL17A is a pro-inflammatory cytokine secreted from activated T-helper cell known as T-helper 17 cell [38]. IL17A^+^ cell was found in CCA intratumoral areas which was correlated with lymph node metastasis, intrahepatic metastasis, and tumor progression to advanced stages [39]. The univariate analyses showed that the increase of IL17A^+^ cells in CCA intratumoral was significantly associated with the shortening of patients’ survival [39]. Plasma concentrations of IL17A were found to be 2.5-fold higher in patients with CCA and long-term liver fluke infection than in patients with liver fluke infection without CCA and healthy control (21-fold higher) [40]. In support of these previous reports, our result showed that IL17A level was significantly decreased at 24 h in subjects receiving multiple doses of AL, compared with placebo. Apart from IL17A, IL10 level appeared to be increased on days 7 and 14 of dosing. IL10 is strongly immunosuppressive cytokines. It not only induces anergy in T cells, but also inhibits the activation of inflammatory reactions by mast cells and eosinophils [41]. Tumor-associated Tregs secretes IL10 and TGF-β, which inhibit cytotoxic T cells and NK cells and shape-up an immunosuppressive milieu [42, 43]. High Treg cell frequency is speculated to reflect a poor prognosis in pancreatic and colon cancer patients [44]. Moreover, IL10 was shown to exert antitumoral activity in gliomas, melanomas, and breast and ovarian carcinomas [45]. Atractylenolide I was reported to enhance IL10 expression in LPS-induced acute lung injury mouse model [31].

Results from earlier studies proposed IFNγ as a prototypical antitumor cytokine. Results from subsequent studies however, supported its role in controlling tumor initiation and progression and in promoting the immunoevasive property. IFNγ pretreatment potentiated lung colonization of intravenously inoculated B16 melanoma due to upregulation of MHC class I molecules on tumor cells and decreased sensitivity to NK cells [46]. In the in vivo study, increasing of metastatic potential was also attributed to the increased resistance to NK cells in IFNγ gene-transfected TS/A mammary adenocarcinoma cells [47]. Moreover, obstruction of bile ducts was shown to be triggered by IFNγ [48]. In the present study, no significant effects of AL on the production of IFNγ as well as IL2, IL4, IL6, and IL10, TNFα were found. This could be due to the normal health condition of the volunteers included in the study. Unlike the in vitro condition of which the production of cytokines was limited to only PBMCs, serum cytokine levels in reflecting the overall systemic cytokine production from several cell types [49]. Cytokine detection in the supernatant of cultured PBMCs, therefore provides more specific cytokine expression from the immune cell, especially the mononuclear cells, which plays a central role in immune response [28]. Furthermore, due to large interindividual variability in serum level of each cytokine, statistical significance was not reached. Further large-scale studies in CCA patients are needed to substantiate the immunomodulatory role of AL.

NK cells can sense and respond to ‘stressed’ cells, such as cancer cells, in the nearby area [50]. Lysis of target cells, including neoplastic cells, is the hallmark function of NK cells and paramount importance for their tumor-inhibiting efficacy [48]. After activation, NK cells secrete several cytokines such as IFNγ, TNFα, granulocyte-macrophage colony-stimulating factor (GM-CSF), and chemokines (CCL1, CCL2, CCL3, CCL4, CCL5, and CXCL8) that can modulate the function of other innate and adaptive immune cells [51]. Antitumor effects of NK cells can be overcome by various approaches, for examples, conservation of MHC class I (MHC-I) expression, shedding of ligands for the activating NK receptor (e.g.*,* NKG2DLs), and secretion of immunomodulatory molecules (e.g.*,* TGF-β, prostaglandin E2, and adenosine) by tumor cells, ultimately resulting in tumor progression [50]. Our study demonstrated a significant increase in NK cells at almost all observation time points (days 3, 7, and 14). Tumor-infiltrating lymphocytes (TILs) are highly heterogeneous population which include B lymphocytes, CD8^+^ cytotoxic T lymphocytes, cytokine-secreting CD4^+^ T helper lymphocytes, and Forkhead box P3 (FoxP3)^+^ Tregs [52]. In CCA, CD8^+^ and CD4^+^ cytotoxic T lymphocytes have been investigated with regard to their presence, locations within the tumor, and association with patients’ survival. Multiple studies confirm that enhanced CD4^+^ and CD8^+^ infiltrates (also in combination with low numbers of macrophages) in CCA and extrahepatic biliary tract cancer are associated with improvement of overall survival, fewer lymph node metastases and reduced venous and perineural invasion [53–57], whereas small amounts of CD8^+^ TILs are associated with poor overall survival [58]. Total tumor-infiltrating B lymphocytes were shown to be correlated with a longer overall survival probability in biliary tract cancer [53]. In our study, significant increase in CD4^+^ in AL-treated subjects compared with placebo was observed on days 7 and 23, and a significant increase in CD8^+^ was observed on day 14 in subjects receiving multiple dosing of AL. Taken together, these results suggested that under normal conditions, oral administration of AL can stimulate the production of innate and adaptive immune cells.

The cytotoxic activity against CL-6 cells of the NK cells in PBMCs appeared to be increased at 24 h of a single or multiple dosing of AL compared with placebo. The previous study reported that the co-culture of CCA cells with the epidermal growth factor receptor monoclonal antibody-- cetuximab, and NK cells significantly enhanced CCA cell death by potentiating antibody-dependent cellular cytotoxicity [59]. Similarly, infusion of ex vivo expanded human NK cells into CCA xenograft mice resulted in inhibition of tumor growth [58]. Notably, among the CCA cell lines, ex vivo expanded human NK cells showed a higher cytolytic activity to HuCCT1 and SNU308, but lower activity in SNU1196 and SNU478 cells [60]. This suggested that NK cells display variable cytolytic effects depending on the various cancer types.

## Conclusion

Results from the present study confirm the immunomodulatory activity of AL in humans. This activity, in complementary with the direct action of AL on inducing CCA cell apoptosis, suggests its potential role for CCA control. Further studies are underway to support the immunomodulatory activity in CCA patients.

## Supplementary Information

**Additional file 1.**

## Data Availability

The datasets generated and/or analysed during the current study are available as supplementary file and additional data are available from the corresponding author on reasonable request.

## Abbreviations

AL: *Atractylodes lancea* (Thunb.) D.C
CAM: Calcein-AM
CBA: Cytometric bead array
CCA: Cholangiocarcinoma
CCL: Chemokine *(C-C motif) ligand*
CD: Cluster of differentiation
cDNA: Complementary deoxyribonucleic acid
CI: Confidence interval
ConA: Concanavalin A
COX-2: Cyclooxygenase-2
CSF2: Colony stimulating factor-2
Ct: Cycle threshold
CXCL: Chemokine (C-X-C motif) ligand
DEPC: Diethyl pyrocarbonate
DMSO: Dimethyl sulfoxide
dNTP: Deoxynucleoside triphosphate
DTT: Dithiothreitol
EDTA: Ethylenediaminetetraacetic acid
EGFR: Epidermal growth factor receptor
EMT: Epithelial‐to‐mesenchymal transition
E:T: Effector to target ratio
FBS: Fetal bovine serum
5-FU: Fluorouracil
GAPDH: glyceraldehyde-3-phosphate dehydrogenase
GM-CSF: Granulocyte-macrophage colony-stimulating factor
HMC-1: Human mast cell line
IC_50_: The concentration that inhibits cell growth by 50%
IFN-: Interferon‐gamma
IL: Interleukin
iNOS: inducible nitric oxide synthase
LPS: Lipopolysaccharide
MAPK: Mitogen-activated protein kinase
Mcl-1: Myeloid cell leukemia-1
MHC: Major histocompatibility complex
mRNA: Messenger ribonucleic acid
MTT: 3-(4,5-dimethylthiazol-2-yl)-2,5-diphenyl tetrazolium bromide
NK: Natural killer
NKG2DL: Natural killer group 2, member D ligand
NO: Nitric oxide
NOS: Nitrous oxide systems
PBMC: Peripheral blood mononuclear cell
PBS: Phosphate buffer saline
PCR: Polymerase chain reaction
PGE2: Prostaglandin E2
PI: Propidium iodide
RNase: Ribonuclease
RPMI: Roswell park memorial institute
RT-PCR: Reverse transcription polymerase chain reaction
SD: Standard deviation
STAT-3: Signal transducer and activator of transcription-3
TAM: Tumor-associated macrophage
TGF‐β: Transforming growth factor-beta
Th1: T helper type 1
TIL: Tumor-infiltrating lymphocyte
TNFα: Tumor necrosis factor-alpha
Treg cell: Regulatory T cell
TS/A: Highly metastatic *mouse mammary adenocarcinoma* cell line
TSLP: Thymic stromal lymphopoietin
VEGF: Vascular endothelial growth factor
WBC: White blood cell
ZEB2: Zinc finger E-box binding homeobox-2
