# Supplementary material for: A randomized placebo-controlled phase I clinical trial to evaluate the immunomodulatory activities of Atractylodes lancea (Thunb) DC. in healthy Thai subjects
Source: BMC Complement Med Ther. 2021 Feb 12;21:61. doi: 10.1186/s12906-020-03199-6 (PMC7879636; doi:10.1186/s12906-020-03199-6)
Supplement: Supplementary file 1 — Additional file 1. [file 12906_2020_3199_MOESM1_ESM.docx]

**Supplemetary 1** Demographics and baseline vital signs of 40 healthy Thai study participants (20 males, 20 females) allocated to *group 1* (a single dose of 1,000 mg capsule formulation of the standardized AL extract) and *group 2* (daily doses of 1,000 mg capsule formulation of the standardized AL extract for 21 days). Data are presented as number or median (range) values.

|  | **Group 1** | | **Group 2** | |
| --- | --- | --- | --- | --- |
|  | **AL** | **Placebo** | **AL** | **Placebo** |
| Male: Female (n) | 10, 10 | 2, 2 | 10, 10 | 2, 2 |
| Age (years) | 22.0 (21.0-27.0) | 23.2 (20.9-27.5) | 25.0 (21.0-29.0) | 23.9 (20.8-28.3) |
| Body weight (kg) | 62.8 (54.9-65.5) | 63.4 (55.5-62.8) | 61.0 (56.7-66.2) | 62.2 (56.2-64.7) |
| Height (cm) | 163.0  (158.0-170.5) | 164.8  (159.5-172.5) | 165.0  (161.0-170.5) | 164.5  (159.0-169.5) |
| BMI | 23.07  (21.1-24.6) | 22.9  (22.2-24.4) | 22.41  (20.74-24.17) | 23.40  (21.55-24.89) |
| Systolic blood pressure (mmHg) | 120 (110-130) | 120 (102-130) | 121 (116-129) | 122 (108-126) |
| Diastolic blood pressure (mmHg) | 67 (60-74) | 66 (60-72) | 69 (62-78) | 66 (62-74) |
| Heart rate (/min) | 69 (61-74) | 70 (64-76) | 72 (65-78) | 71 (62-74) |
| Respiratory rate (/min) | 22 (20-22) | 22 (20-22) | 22 (21-22) | 21 (10-22) |
| Body temperature (^o^C) | 36.5 (36.4-36.7) | 36.6 (36.2-37.0) | 36.6 (36.5-36.8) | 36.3 (36.4-36.9) |

**Supplemetray 2** Laboratory data of 40 healthy Thai study participants (20 males, 20 females) allocated to *group 1* (a single dose of 1,000 mg capsule formulation of the standardized AL extract) and *group 2* (daily doses of 1,000 mg capsule formulation of the standardized AL extract for 21 days). Data are presented as median (range) or percentage (%) values.

1. **Hematology**

| **Parameters** | **Normal range** | **Group 1** | | | | **Group 2** | | | |
| --- | --- | --- | --- | --- | --- | --- | --- | --- | --- |
|  |  | **Placebo**  **(n=4)**  **Day 0** | **AL (n=20)** | | | **Placebo**  **(n=4)**  **Day 0** | **AL (n=20)** | | |
|  |  |  | **Day 0** | **Day 4** | **Day 14** |  | **Day 0** | **Day 4** | **Day 21** |
| WBC (x10^3^/μl) | 4.0-11.0 | 6.02  (5.05-7.23) | 6.31  (5.55-7.30) | 5.99  (5.70-7.30) | 5.97  (4.92-7.54) | 6.02  (5.11-7.09) | 6.25  (5.05-7.24) | 5.99  (4.91-6.44) | 6.16  (4.53-6.68) |
| Platelets (x10^3^/μl) | 150-400 | 249.5  (215-286) | 252.5  (217-279) | 251.5  (216-279) | 255  (241-306) | 266.0  (245-282) | 261.0  (221-279) | 262.5  (251-293) | 270  (245-314) |
| RBC (x10^6^/μl)^a^ | 4.5-6.0 | 5.02  (4.30-5.48) | 5.10  (4.47-5.52) | 4.91  (4.43-5.30) | 4.94  (4.08-5.52) | 4.80  (4.60-5.03) | 4.99  (4.69-5.23) | 4.82  (4.64-4.92) | 4.70  (4.33-5.10) |
| Hemoglobin (mg/dL)^b^ | Male:14.0-18.0  Female: 12.0-16.0 | 13.9  (12.9-14.8) | 13.8  (13.1-14.6) | 13.65  (12.0-14.3) | 13.5  (11.9-14.4) | 14.2  (13.0-14.9) | 14.4  (13.0-14.9) | 13.5  (12.4-14.3) | 13.1  (12.2-14.6) |
| Hematocrit (%)^c^ | Male:39.0-57.0  Female: 36.0-48.0 | 42.22  (38.8-44.9) | 42.15  (39.6-44.2) | 41.6  (39.1-45.4) | 42.5  (36.8-44.7) | 42.12  (39.2-45.8) | 43.25  (39.6-46.0) | 41.1  (38.9-44.2) | 41.5  (38.5-45.3) |
| Neutrophil (%) | 45-75 | 55.9  (52.5-58.0) | 54.4  (52.5-58.0) | 54.7  (47.6-59.8) | 57.0  (51.9-62.3) | 54.2  (50.8-56.6) | 55.2  (52.4-59.9) | 54.75  (51.7-57.6) | 53.30  (48.9-57.3) |
| Lymphocyte (%) | 20-45 | 37.3  (33.9-40.6) | 37.5  (34.1-41.3) | 37.2  (33.2-41.7) | 34.6  (31.6-39.7) | 37.55  (33.2-40.1) | 37.35  (32.2-41.7) | 38.65  (35.1-41.0) | 38.00  (35.4-40.7) |
| Monocyte (%) | 2.0-10.0 | 3.50  (3.20-4.20) | 3.80  (3.30-4.60) | 3.60  (3.0-4.3) | 3.6  (2.9-4.5) | 3.20  (3.2-4.3) | 3.30  (3.0-4.1) | 3.25  (3.2-4.7) | 3.10  (3.2-4.9) |
| Eosinophil (%) | 4.0-6.0 | 2.55  (1.70-5.20) | 2.75  (1.60-5.50) | 3.65  (2.0-6.1) | 2.7  (2.1-5.5) | 2.89  (2.5-3.8) | 3.00  (2.3-4.0) | 2.85  (2.5-4.0) | 3.70  (2.9-3.7) |
| Basophil (%) | 0.0-1.0 | 0.4  (0.3-0.5) | 0.4  (0.4-0.5) | 0.4  (0.3-0.5) | 0.4  (0.3-0.5) | 0.35  (0.3-0.5) | 0.35  (0.3-0.4) | 0.35  (0.3-0.5) | 0.40  (0.3-0.5) |
| PT (sec) | 10.2-12.6 | 11.25  (11.0-12.2) | 11.85  (11.3-12.0) | 11.45  (11.1-11.8) | 11.5  (11.3-11.7) | 11.16  (10.8-11.5) | 11.2  (11.0-11.9) | 11.15  (10.9-11.7) | 11.0  (10.6-11.6) |
| INR (sec) | 2-3 | 1.002  (0.93-1.01 | 1.005  (0.95-1.02) | 0.965  (0.94-1.00) | 0.97  (0.95-0.99) | 0.94  (0.91-1.02) | 0.945  (0.93-1.01) | 0.945  (0.92-0.99) | 0.93  (0.89-0.98) |
| PTT (sec) | 22.2-28.3 | 26.1  (25.5-28.6) | 26.4  (25.9-29.1) | 26.2  (24.9-27.5) | 26.0  (24.4-27.5) | 25.4  (24.2-27.5 ) | 25.9  (24.3-27.9 ) | 25.5  (24.0-27.8) | 24.0  (22.6-26.2) |

*^a^ Statistical significant difference: Group 2 between Day 4 vs. Day 0 (p=0.01) and Day 21 vs. Day 0 (p=0.02, Wilconxon Sign-Rank test)*

*^b^ Statistical significant difference: Group 2 between Day 4 vs. Day 0 (p=0.01) and Day 21 vs. Day 0 (0.02, Wilcoxon Signed Rank test)*

*^c^ Statistical significant difference: Group 2 between Day 4 vs Day 0 (p=0.03)and Day 21 vs. Day 0 (p=0.019, Wilcoxon Signed Rank test)*

1. **Biochemistry**

| **Parameters** | **Normal range** | **Group 1** | | | | **Group 2** | | | |
| --- | --- | --- | --- | --- | --- | --- | --- | --- | --- |
|  |  | **Placebo**  **(n=4)**  **Day 0** | **AL (N=20)** | | | **Placebo**  **(n=4)**  **Day 0** | **AL (n=20)** | | |
|  |  |  | **Day 0** | **Day 4** | **Day 14** |  | **Day 0** | **Day 4** | **Day 21** |
| Creatinine (mg/dl) | 0.67-1.17 | 0.85  (0.70-0.99) | 0.84  (0.73-1.03) | 0.88  (0.73-0.97) | 0.87  (0.74-0.97) | 0.84  (0.75-1.06) | 0.82  (0.73-0.99) | 0.83  (0.74-0.90) | 0.87  (0.79-1.07) |
| BUN (mg/dl) | 7-18 | 12.40  (10.5-13.3) | 12.55  (10.8-12.3) | 13.00  (10.5-14.9) | 12.90  (9.8-15.1) | 12.46  (9.5-13.0) | 12.5  (9.9-13.6) | 12.45  (9.9-13.5) | 12.4  (9.9-13.0) |
| Uric acid (mg/dl) | 3.7-7.2 | 4.70  (3.70-5.50) | 4.80  (3.80-5.70) | 4.65  (3.70-5.60) | 4.50  (3.80-6.10) | 4.80  (3.82-5.70) | 4.85  (3.80-5.90) | 4.80  (3.80-5.80) | 4.80  (3.80-6.10) |
| AST (U/l) | 15-37 | 16.5  (15.5-20.0) | 16.0  (15.0-18.0) | 16.5  (15.0-22.0) | 15.0  (16.0-20.0) | 14.5  (15.0-18.0) | 14.0  (15.0-18.0) | 15.5  (16.0-19.0) | 17.0  (16.0-21.0) |
| ALT (U/l) | 16-63 | 27.0  (18.0-35.0) | 30.0  (20.0-39.0) | 27.5  (20.0-35.5) | 25.0  (20.0-33.0) | 28.0  (18.5-34.0) | 28.5  (19.0-33.0) | 30.0  (22.0-35.0) | 29.0  (24.0-37.0) |
| Direct Bilirubin (mg/dl) | 0.0-0.20 | 0.18  (0.20-0.20) | 0.20  (0.20-0.20) | 0.20  (0.10-0.20) | 0.18  (0.10-0.20) | 0.10  (0.10-0.20) | 0.10  (0.10-0.20) | 0.10  (0.10-0.20) | 0.10  (0.10-0.18) |
| Total Bilirubin (mg/dl) | 0.2-1.00 | 0.65  (0.50-0.80) | 0.65  (0.60-0.90) | 0.60  (0.40-0.60) | 0.60  (0.40-0.90) | 0.52  (0.43-0.65) | 0.55  (0.40-0.70) | 0.50  (0.40-0.60) | 0.50  (0.40-0.60) |
| Total Protein (mg/dl) | 6.4-8.2 | 7.65  (7.60-8.30) | 7.95  (7.70-8.20) | 7.70  (7.50-8.20) | 7.70  (7.60-7.90) | 7.80  (7.40-8.10) | 7.90  (7.50-8.30) | 7.80  (7.30-8.10) | 7.70  (7.50-8.00) |
| Albumin (mg/dl) | 3.5-4.0 | 4.15  (3.90-4.26) | 4.20  (4.00-4.30) | 4.10  (3.90-4.30) | 4.10  (3.90-4.30) | 4.10  (4.00-4.50) | 4.20  (4.00-4.40) | 4.00  (3.90-4.30) | 4.00  (3.80-4.40) |
| LDH (U/l) | 207-414 | 310.5  (299-330) | 317  (300-336) | 311.5  (298-362) | 309  (295-364) | 300.5  (282-332) | 297.5  (274-332) | 302.0  (278-323) | 292.0  (268-316) |
| ALP (U/l) | 46-116 | 58.0  (50.0-78.0) | 60.5  (53.0-71.0) | 57.5  (49.0-70.0) | 61.0  (48.0-70.0) | 58.5  (50.5-70.0) | 58.0  (50.0-73.0) | 58.5  (49.0-73.0) | 59.0  (49.0-70.0) |
| CPK (U/l) | 38-174 | 139.5  (101.0-227.0) | 140.5  (89.0-227.0) | 140.5  (93.0-296.0) | 139.0  (103.0-296.0) | 132.5  (90.0-169.0) | 130.0  (89.0-165.0) | 133.5  (83.0-168.0) | 135.0  (86.0-177.0) |
| Phosphorus (mg/dl) | 2.5-4.9 | 4.0  (3.8-4.2) | 4.0  (3.7-4.1) | Not applicable | Not applicable | 3.80  (3.70-4.10) | 3.80  (3.70-4.30) | Not applicable | Not applicable |
| Calcium (mEq/l) | 8.5-10.1 | 9.60  (9.40-9.50) | 9.70  (9.60-10.00) | Not applicable | Not applicable | 9.70  (9.20-10.00) | 9.75  (9.50-10.00) | Not applicable | Not applicable |
| Total cholesterol (mg/dl) | 0-200 | 200  (170-220) | 203  (177-231) | 202  (171-217) | 210  (165-221) | 203  (182-220) | 203.5  (192-211) | 196.5  (185-213) | 207.0  (193-242) |
| Triglycerides (mg/dl) | 0-150 | 63.0  (40.0-70.0) | 62.0  (43.0-72.0) | 60.5  (44.0-79.0) | 65.0  (46.0-92.0) | 63.5  (43.5-90.5) | 64.0  (43.0-93.0) | 64.0  (48.0-95.0) | 67.0  (47.0-105.0) |
| LDL (mg/dl) | 0-100 | 127.0  (112.0-155.0) | 127.0  (112.0-155.0) | 128.5  (115.0-142.0) | 127.0  (103.0-152.0) | 124.0  (114.0-140.0) | 124.5  (118.0-139.0) | 123.5  (112.0-145.0) | 130.0  (115.0-145.0) |
| HDL (mg/dl) | 40-60 |  | 61.0  (55.0-73.0) | 61.5  (51.0-68.0) | 60.0  (52.0-69.0) | 60.5  (53.9-67.9) | 60.5  (52.0-68.0) | 59.0  (55.0-65.0) | 61.0  (54.0-67.0) |

1. **Urinalysis**

| **Parameters** | **Normal**  **range** | **Group 1** | | | | **Group 2** | | | |
| --- | --- | --- | --- | --- | --- | --- | --- | --- | --- |
|  |  | **Placebo**  **(n=4)**  **Day 0** | **AL (n=20)** | | | **Placebo**  **(n=4)**  **Day 0** | **AL (n=20)** | | |
|  |  |  | **Day 0** | **Day 4** | **Day 14** |  | **Day 0** | **Day 4** | **Day 21** |
| Specific gravity | 1.003-1.029 | 1.018  (1.015-1.024) | 1.020  (1.016-1.025) | 1.020  (1.015-1.025) | 1.015  (1.010-1.020) | 1.020  (1.018-1.026) | 1.020  (1.020-1.025) | 1.020  (1.015-1.025) | 1.020  (1.015-1.023) |
| pH | 4.5-7.8 | 6.3  (6.0-7.0) | 6.5  (6.0-7.0) | 6.0  (6.0-7.0) | 6.0  (5.5-6.5) | 6.3  (6.0-7.0) | 6.5  (6.0-7.0) | 6.5  (6.0-7.0) | 6.5  (6.0-7.0) |
| Protein:  Negative (%)  Trace (%)  1+ (%)  2+ (%) | Negative/  Trace | 100%  0  0  0 | 100%  0  0  0 | 35  64  0  0 | 74  26  0  0 | 90  10  0  0 | 95  5  0  0 | 90  10  0  0 | 74  25  0  0 |
| Glucose:  Negative (%) | Negative | 100 | 100 | 100 | 100 | 100 | 100 | 100 | 100 |
| Ketones:  Negative (%)  Trace (%)  1+ (%)  2+ (%) | Negative | 100  0  0  0 | 100  0  0  0 | 100  0  0  0 | 100  0  0  0 | 100  0  0  0 | 100  0  0  0 | 100  0  0  0 | 100  0  0  0 |

**Supplementary 3** ECG data of 40 healthy Thai study participants (20 males, 20 females) allocated to *group 1* (a single dose of 1,000 mg capsule formulation of the standardized AL extract) and *group 2* (daily doses of 1,000 mg capsule formulation of the standardized AL extract for 21 days). Data are presented as median (range) or percentage (%) values.

|  | **Normal range** | **Palcebo**  **(n=4)**  **Day 0**  **H 0** | **AL (n=20)** | | | | | | |
| --- | --- | --- | --- | --- | --- | --- | --- | --- | --- |
|  |  |  | **Day 0** | | | | **Day 5** | **Day 7** | **Day 14** |
|  |  |  | **H 0** | **H 2** | **H 6** | **H 12** |  |  |  |
| Ventricular rate (bpm) | 60-100 | 64  (62-76) | 63  (60-72) | 63.5  (62-76) | 63  (59-68) | 63  (64-68) | 65  (62-70) | 63  (62-68) | 64.5  (62-72) |
| RR interval (msec) | 600-1200 | 940  (870-1040) | 938  (846-1043) | 931  (908-1014) | 949  (874-1010) | 949  (887-1012) | 914  (852-1049) | 973  (879-1059) | 998  (830-1300) |
| PR interval  (msec) | 120-200 | 156  (140-174) | 152  (136-162) | 155  (146-170) | 151  (138-164) | 156  (144-166) | 150  (144-164) | 150  (140-166) | 156  (128-166) |
| QRS interval  (msec) | 80-100 | 88  (80-90) | 90  (88-94) | 90  (80-94) | 89  (84-96) | 89  (84-94) | 90  (86-96) | 91  (88-96) | 90  (88-96) |
| QTc interval  (msec) | < 430 | 420  (390-410) | 419  (396-430) | 420.5  (404-430) | 415  (403-430) | 413  (404-423) | 419  (417-430) | 414.5  (402-429) | 414  (381-438) |

**Supplementary 4** ECG data of 20 healthy 40 healthy Thai study participants (20 males, 20 females) allocated to *group 1* (a single dose of 1,000 mg capsule formulation of the standardized AL extract) and *group 2* (daily doses of 1,000 mg capsule formulation of the standardized AL extract for 21 days). Data are presented as median (range) or percentage (%) values. Data are presented as median (range) or percentage (%) values.

| **Parameters** | **Normal range** | **Placebo**  **(n=4)**  **Day 0**  **H 0** | **AL (n=20)** | | | | | | |
| --- | --- | --- | --- | --- | --- | --- | --- | --- | --- |
|  |  |  | **Day 0** | | | | **Day 1** | **Day 2** | **Day 5** |
|  |  |  | **H0** | **H2** | **H6** | **H12** |  |  |  |
| Ventricular rate (bpm) | 60-100 | 67.0  (64-78) | 67.5  (66-75) | 65.5  (67-71) | 66  (65-73) | 65  (62-68) | 63  (64-70) | 67.5  (62-74) | 73.5  (63-79) |
| RR interval (msec) | 600-1200 | 910.0  (890-1020) | 876.5  (789-984) | 937  (839-1041) | 898.5  (812-1005) | 919  (872-984) | 942  (877-1042) | 865  (799-959) | 813  (753-951) |
| PR interval  (msec) | 120-200 | 144.2  (138-156) | 144.5  (140-154) | 149  (140-162) | 148  (144-160) | 146  (140-154) | 146  (142-158) | 148  (140-162) | 145  (140-160) |
| QRS interval  (msec) | 80-100 | 90  (89-92) | 94  (86-96) | 89  (84-96) | 92  (88-98) | 91  (84-100) | 93  (84-98) | 95  (86-100) | 93  (88-101) |
| QTc interval  (msec) | < 430 | 414  (410-428) | 414  (410-428) | 408  (412-422) | 408.5  (412-426) | 408  (410-427) | 419^a^  (407-443) | 406.5  (410-431) | 407  (409-429) |

|  | **Normal range** | **Day 7** | **Day 9** | **Day 11** | **Day 14** | **Day 18** | **Day 20** |
| --- | --- | --- | --- | --- | --- | --- | --- |
| Ventricular rate (bpm) | 60-100 | 69  (62-76) | 66.5  (62-77) | 68  (62-75) | 69  (63-77) | 68  (63-76) | 72  (64-80) |
| RR interval (msec) | 600-1200 | 866  (782-982) | 895  (779-961) | 881  (796-996) | 865  (773-944) | 875  (783-974) | 825  (745-976) |
| PR interval  (msec) | 120-200 | 152  (144-166) | 148  (134-158) | 150  (140-162) | 148  (138-160) | 154  (136-162) | 152  (144-162) |
| QRS interval  (msec) | 80-100 | 98  (88-104) | 94  (88-100) | 94  (88-100) | 96  (90-98) | 96  (92-98) | 94  (86-100) |
| QTc interval  (msec) | < 430 | 414  (412-423) | 410  (401-421) | 409  (407-417) | 410  (405-413) | 411  (401-419) | 412  (404-416) |

|  | **Normal range** | **Day 21** | | | | **Day 22** | **Day 23** |
| --- | --- | --- | --- | --- | --- | --- | --- |
|  |  | **H0** | **H2** | **H6** | **H12** |  |  |
| Ventricular rate (bpm) | 60-100 | 72  (65-76) | 63  (58-69) | 66  (59-74) | 65  (60-71) | 61  (59-72) | 65  (61-78) |
| RR interval (msec) | 600-1200 | 828  (783-921) | 940  (867-1031) | 903  (805-1013) | 914  (840-991) | 968  (826-1006) | 918  (763-981) |
| PR interval  (msec) | 120-200 | 150  (140-164) | 152  (140-164) | 150  (138-164) | 150  (140-156) | 150  (138-164) | 150  (138-160) |
| QRS interval  (msec) | 80-100 | 96  (88-100) | 90  (88-100) | 94  (88-98) | 90  (88-98) | 96  (88-100) | 96  (88-102) |
| QTc interval  (msec) | < 400 | 408  (402-420) | 408  (401-420) | 408  (402-417) | 404  (402-421) | 414  (402-421) | 403  (402-417) |
